# Understanding the Variability of Certain Biological Properties of H1N1pdm09 Influenza Viruses

**DOI:** 10.3390/vaccines10030395

**Published:** 2022-03-03

**Authors:** Mohammad Al Farroukh, Irina Kiseleva, Ekaterina Bazhenova, Ekaterina Stepanova, Ludmila Puchkova, Larisa Rudenko

**Affiliations:** 1 Federal State Budgetary Scientific Institution “Institute of Experimental Medicine”, 197376 St. Petersburg, Russia; sonya.01.08@mail.ru (E.B.); fedorova.iem@gmail.com (E.S.); puchkovalv@yandex.ru (L.P.); vaccine@mail.ru (L.R.); 2 Peter the Great St. Petersburg Polytechnic University, Institute of Biomedical Systems and Biotechnology, Graduate School of Biomedical Systems and Technologies, 195251 St. Petersburg, Russia

**Keywords:** A(H1N1)pdm09 influenza viruses, evolution, viral toxicity, viral pathogenicity, thermal stability of the hemagglutinin, temperature sensitivity of reproduction, polymerase complex

## Abstract

The influenza virus continually evolves because of the high mutation rate, resulting in dramatic changes in its pathogenicity and other biological properties. This study aimed to evaluate the evolution of certain essential properties, understand the connections between them, and find the molecular basis for the manifestation of these properties. To that end, 21 A(H1N1)pdm09 influenza viruses were tested for their pathogenicity and toxicity in a mouse model with a *ts/non-ts* phenotype manifestation and HA thermal stability. The results demonstrated that, for a strain to have high pathogenicity, it must express a toxic effect, have a *non-ts* phenotype, and have a thermally stable HA. The ancestor A/California/07/2009 (H1N1)pdm influenza virus expressed the *non-ts* phenotype, after which the cycling trend of the *ts/non-ts* phenotype was observed in new strains of A(H1N1)pdm09 influenza viruses, indicating that the ratio of the *ts* phenotype will increase in the coming years. Of the 21 tested viruses, A/South Africa/3626/2013 had the high pathogenicity in the mouse model. Sequence alignment analysis showed that this virus has three unique mutations in the polymerase complex, two of which are in the PB2 gene and one that is in the PB1 gene. Further study of these mutations might explain the distinguishing pathogenicity.

## 1. Introduction

The influenza virus is a worldwide disease that affects up to 5–15% of the global population. The mortality from influenza-associated respiratory disease is estimated to be more than half a million each year, and that number significantly increases when a new strain emerges to cause a pandemic [1]. The first known influenza pandemic was the Spanish flu (1918–1920, death toll ~17.4 million) [2], the second was the Asian flu (1957–1958, death toll 1–4 million), the third was the Hong Kong flu (1968–1969, death toll 1–4 million), [3] and after that came the Russian flu (1977–1979, over 700,000 deaths) [4]. The last pandemic was influenza A(H1N1) in 2009–2010, which is estimated to have caused about 400,000 deaths in 2009 alone [5]. To date, it is impossible to know when influenza pandemics are going to arise, which makes evolutionary studies of the influenza virus highly important. Influenza is an RNA virus that is part of the *Orthomyxoviruses* family, which is divided into four subfamilies: A, B, C, and D. Influenza A is also divided into several subtypes depending on the combination of the hemagglutinin protein (HA), which has 18 subtypes, and the neuraminidase protein (NA), which has 11 subtypes [6]. This study will focus on the evolution of the H1N1pdm09 pandemic of the virus. 

There are three essential properties that an influenza virus strain must have to cause a pandemic: firstly, the strain must not be previously recognized by human immunity; secondly, the strain should have high pathogenicity for the human, and finally, the virus strain should be transmissible and able to cause infection on person-to-person interaction [7]. In 2009, a new A(H1N1)pdm09 influenza virus strain appeared that had all of the three previously mentioned properties. The new pandemic influenza virus had numerous biological features typical of pandemic viruses; in particular, it had pronounced *non-ts* and *non-ca* phenotypes and resistance to sera non-specific inhibitors [8].

The influenza virus has a high mutation rate, which is the basis for the evolutionary variability of the influenza virus [9]. One cycle of virus replication may lead to a new strain with significant changes in its viral biocharacteristics and pathogenicity. A comparative analysis of essential biological properties of evolving H1 viruses has not yet been carried out. Of these characteristics, the HA thermal stability, toxicity, and *non-ts* phenotype warrant the most attention. A *Non-ts* phenotype is the virus’ ability to reproduce at elevated temperatures of the low respiratory tract [10,11,12,13]. For human influenza A viruses, this ability might be inherited from their ancestor that replicated in animal hosts (birds or pigs) that have higher physiological body temperatures (40–42 °C) [14]. Some of the influenza viruses are characterized as lethal in animal models two to four days after inoculation with a high dose of the viral particles [15,16,17]; this characteristic is referred to as viral toxicity. HA stability is the ability of the hemagglutinin protein to resist denaturation and maintain its function in stress conditions such as elevated temperature [18]. The evolution of and changes in these characteristics result from mutations acquired in the viral genome.

The influenza A virus contains eight negative-sense, single-stranded viral RNA gene segments, which encode the 10 essential viral proteins (HA, NA, M2, M1, NP, NS1, NS2, PB1, PB2, and PA). A mutation in any of these proteins might change the viral characteristic and thus affect its pathogenicity. The heterotrimeric polymerase complex, consisting of three subunits—PB1 (polymerase basic protein 1), PB2 (polymerase basic protein 2), and PA (polymerase acidic protein)—has gained the most interest [19]. Each of these subunits contributes to polymerase function: the PA snitches the Cap of cellular mRNA through its endonuclease domain [20], while the PB2’s functions are mRNA recognition and binding of “cap” structures and the PB1 participates in transcriptase primer elongation and endonuclease activity [21]. In addition, the polymerase complex has a significant effect on the manifestation of the *ts/non-ts* phenotype. Several studies showed that mutations in the complex can dramatically affect and change the properties of the virus [22,23,24,25].

In this study, we focused on the evolution of these characteristics and the contribution of each of these to the viral pathogenicity; furthermore, we attempted to explain the relationships between the viral properties and determine how they are connected to the evolution of the viral genome.

## 2. Materials and Methods

*Viruses.* The following 21 A(H1N1)pdm09 influenza viruses that circulated in 2009–2020 and four control A(H1N1) influenza viruses, which are currently considered antigenically obsolete, were used in this work, as indicated in Table 1.

The viruses were inoculated in the chorioallantoic cavity of 10–11-day-old chicken eggs purchased from the Sinyavino poultry farm (Kirovsk Area, Leningrad region, Russia). After 48 h of incubation at 32 °C, the viruses were harvested and stored at −70 °C. Following biosafety instructions, the influenza viruses were handled in a biosafety level-2 (BSL-2) laboratory.

*Temperature sensitivity (non-ts phenotype).* The phenotype of virus reproduction was evaluated in developing chicken eggs (10–11 days old) by comparing the virus infectivity at 32 °C and 40 °C, as described in [8]. The log_10_ EID_50_/mL calculation was based on the Reed and Muench method [27]. In this study, data are presented as the relative % titer. Optimal growth was measured at 32 °C (nominal value of 100%) for each virus. Titers for the temperature of 40 °C were calculated as a percentage relative to the 100% maximal value. The reduction of viral infectivity at 40 °C compared to 32 °C was calculated according to the following formula:

Relative % titer = (M40/M32) × 100

where M40 and M32 are infection titers of the virus expressed in log_10_ EID_50_/mL at the 40 °C and 32 °C temperatures of incubation, respectively. The titers of the viruses were calculated based on [27].

*Non-ts*-phenotype viruses have a relative % titer above 60%, while *ts*-phenotype viruses have a relative % titer under 40%. The range from 40% to 60% is an intermediate phase where viruses do not have a clear phenotype.

*Thermal stability of HA.* To measure the thermal stability of HA, infectious allantoic fluid was diluted 1:5 by phosphate-buffered saline (PBS), as described in [18,28]. The viruses were incubated in a Thermo block for inactivation for 20 min at temperatures ranging from 37 to 70 °C. Subsequently, a hemagglutination assay using 1% chicken RBCs was performed. In parallel, control samples were incubated at RT for 20 min. HA was considered to have low stability when it lost activity below 58 °C, above which HA has been considered highly stable as it keeps its activity. 

*Mice.* The study population (880 female CBA mice, aged 8–12 weeks, body mass 18–22 g) was purchased from the laboratory breeding nursery Rappolovo (Leningrad region, Russia). The animals were kept at room temperature (23–25 °C) in cages with wood shavings, the humidity was maintained at 60%, the day-night cycle was 12 h each, and the animals had free access to food and water.

*Toxicity study in mice (development of acute pulmonary edema).* Study groups of 10 mice each were lightly anesthetized with ether, after which a high dose of fresh, non-diluted virus was administered into the nasal fossae. The mice were observed daily during the first six days post-infection to detect mortality from acute pulmonary edema, as previously described in [29]. To consider the virus capable of causing high toxicity, the mortality should be over 50%. However, if the virus causes mortality of 50% or less, it is considered low-level toxic, and if it does not cause mortality, then it is a non-toxic virus.

*Pathogenicity study in mice.* For each tested virus, 10-fold serial dilutions were prepared using PBS. Study groups of 10 mice each were lightly anesthetized with ether, then inoculated with 50 μL of PBS, divided equally into the nasal fossae. Mice mortality from pneumonia was observed for the first 14 days post-infection and the LD_50_ for each virus was calculated by the routine Reed and Muench method [27] and expressed as the log EID_50_/mL required to give 1 LD_50_. Viruses with an LD_50_ < 1.5 were considered non-pathogenic, whereas viruses with an LD_50_ ≥ 4 were considered to have high pathogenicity, and viruses in the interval between 1.5 and 4 were considered capable of causing low pathogenicity. The parameters based on which the toxicological or pathogenic capabilities of the tested viruses were evaluated are given in Table 2. 

In addition, for the A/South Africa/3626/2013 virus, on day 3 post-infection, viral RNA was extracted from mice lung tissue to be sequenced.

The mice and chicken embryos were handled according to the guidelines of the Declaration of Helsinki, European Union legislation, and the Russian Manual for Laboratory Animals [30,31,32]. At the end of the study, the animals were humanely euthanized. The fertilized eggs used for virus propagation were discarded appropriately, according to the Russian Sanitary and Epidemiological Requirements for the Prevention of Infectious Diseases SP 3.3686-21 (approved 28 January 2021) [33].

*Genetic analysis and phylogenetic trees.* The multiple alignment method (MUSCLE algorithm) was used to perform the genetic analysis, and the PHYLIP neighbor-joining method was used to build the phylogenetic trees. Both methods were carried out using the Unipro UGENE v1.12.1 program (https://doi.org/10.1093/bioinformatics/bts091, accessed on 2 December 2021), and all protein sequences were obtained from the GISAID platform.

Full genome sequencing was performed using an ABI Prism 3031xl Genetic Analyzer (Applied Biosystems, Applera Corporation|Foster City, CA 94404, USA) with a BigDye™ Terminator v3.1 Cycle Sequencing Kit (Applied Biosystems, Life Technologies Corporation|Carlsbad, CA 92008, USA ), according to the manufacturer’s instructions. 

Pyrosequencing detection of a single nucleotide polymorphism was carried out using a PyroMark Q24 (QIAGEN, MD, USA) genetic analysis system according to the manufacturer’s instructions. The oligonucleotide ACGGGCAATCTCCAAACA was used for the pyrosequencing reaction.

*Statistics* were analyzed using GraphPad Prism 7. A *p*-value < 0.05 was considered statistically significant.

## 3. Results

### 3.1. Toxicity for Mice

The ancestor of all A(H1N1)pdm09 viruses—the A/California/7/2009 strain—was non-toxic and non-pathogenic for mice. Of the 21 viruses, 11 (52.4%) were highly toxic, where the toxicity results of these viruses were as follows: virus 2, A/Bolivia/559/2013 was 100%; virus 5, A/South Africa/3626/2013 was 80%; virus 6, A/Florida/62/2014 was 80%; virus 7, A/Laos/1187/2014 was 100%; virus 8, A/New York/61/2015 was 60%; virus 10, A/Bangladesh/3002/2015 was 100%; virus 11, A/Newcastle/67/2017 was 80%; virus 12, A/South Australia/272/2017 was 90%; virus 17, A/lowa/12/2019 was 100%; virus 19, A/Guangdong-Maonan/SWL1536/2019 was 90%; and virus 20, A/Arkansas/08/2020 was 80%. Besides this, 10 (47.6%) were non-toxic or slightly toxic (Table 3). 

### 3.2. Pathogenicity for Mice

Of the 21 viruses, only one (4.8%) turned out to be highly pathogenic for mice (A/South Africa/3626/2013). Five of the remaining viruses (23.8%) were slightly pathogenic (virus 2, A/Bolivia/559/2013; virus 4, A/New Hampshire/04/2013; virus 6, A/Florida/62/2014; virus 7, A/Laos/1187/2014; virus 10, A/Bangladesh/3002/2015). Fifteen (71.4%) were non-pathogenic (virus 1, A/California/07/2009; virus 3, A/Mississippi/10/2013; virus 8, A/New York/61/2015; virus 9, A/Slovenia/2903/2015; virus 11, A/Newcastle/67/2017; virus 12, A/South Australia/272/2017; virus 13, A/New Jersey/13/2018; virus 14, A/Darwin/123/2018; virus 15, A/Brisbane/02/2018; virus 16, A/lowa/59/2018; virus 17, A/lowa/12/2019; virus 18, A/Victoria/2570/2019; virus 19, A/Guangdong-Maonan/SWL1536/2019; virus 20, A/Arkansas/08/2020; virus 21, A/Indiana/02/2020; Table 3).

**Figure 1:**
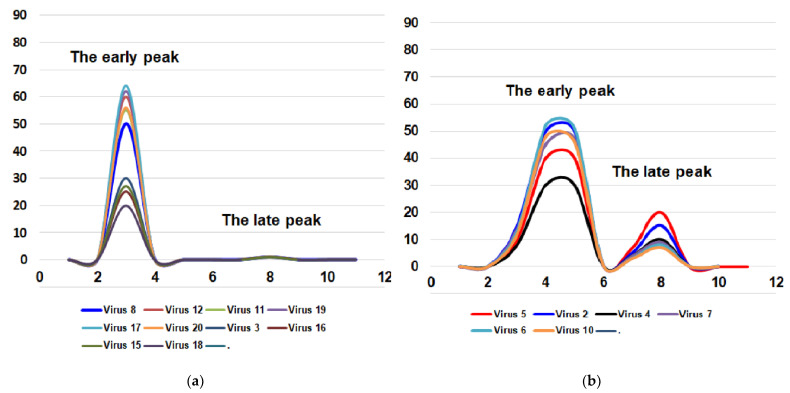

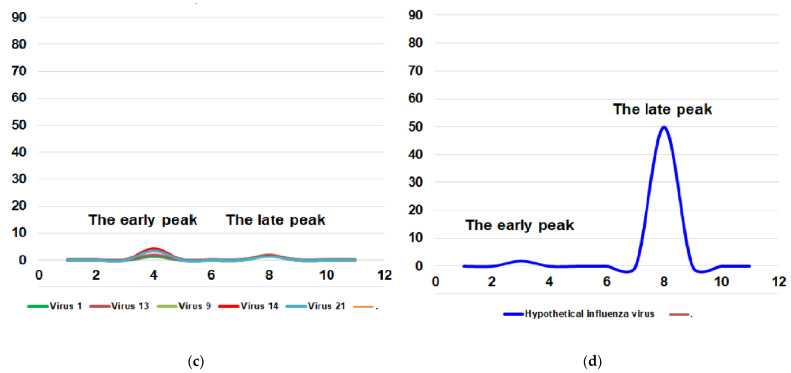
Four possible combinations of toxicity and pathogenicity of influenza virus for mice. Virus 1—A/California/07/2009; virus 2—A/Bolivia/559/2013; virus 3—A/Mississippi/10/2013; virus 4—A/New Hampshire/04/2013; virus 5—A/South Africa/3626/2013; virus 6—A/Florida/62/2014; virus 7—A/Laos/1187/2014; virus 8—A/New York/61/2015; virus 9—A/Slovenia/2903/2015; virus 10—A/Bangladesh/3002/2015; virus 11—A/Newcastle/67/2017; virus 12—A/South Australia/272/2017; virus 13—A/New Jersey/13/2018; virus 14—A/Darwin/123/2018; virus 15—A/Brisbane/02/2018; virus 16—A/lowa/59/2018; virus 17—A/lowa/12/2019; virus 18—A/Victoria/2570/2019; virus 19—A/Guangdong-Maonan/SWL1536/2019; virus 20—A/Arkansas/08/2020; virus 21—A/Indiana/02/2020. X-axis—days post-infection. Y-axis—lethality, %. (**a**) the virus is toxic but not pathogenic for mice; (**b**) the virus is toxic and pathogenic for mice; (**c**) the virus is non-toxic and non-pathogenic for mice; (**d**) the virus is non-toxic but pathogenic for mice. The fourth variant is possible only theoretically. So far, we have not encountered strains with a similar combination of features. As for the other three variants, all the viruses studied were distributed as follows. Group (iii) includes five viruses: A/California/7/2009, A/Slovenia/2903/2015, A/New Jersey/13/2018, A/Darwin/123/2018, and A/Indiana/02/20. Group (ii) included six viruses: A/South Africa/3626/2013, A/Bolivia/559/2013, A/New Hampshire/04/2013, A/Laos/1187/2014, A/Florida/62/2014 and A/Bangladesh/3002/2015 and the first group (i) which has most of the viruses (consisted of ten viruses): A/Mississippi/10/2013, A/New York/61/2015, A/South Australia/272/2017, A/Newcastle/67/2017, A/Iowa/59/2018, A/Brisbane/02/2018, A/Victoria/2570/2019, A/Guangdong-Maonan/SWL1536/2019, A/Iowa/12/2019 and A/Arkansas/08/2020.

For these two important biological features of the influenza virus (toxicity and pathogenicity for mice), it is possible to imagine four combinations: (i) the virus is toxic but non-pathogenic for mice (the most common variant); (ii) the virus is toxic and pathogenic for mice; (iii) the virus is non-toxic and non-pathogenic for mice; (iv) the virus is non-toxic or very slightly toxic and pathogenic for mice (Figure 1).

### 3.3. Temperature Sensitivity of Reproduction of A(H1N1)pdm09 Influenza Viruses (ts Phenotype)

The viral titer at the optimum temperature of 32 °C varied from 6.9 log_10_ EID_50_/mL (A/lowa/12/2019) to 9.2 log_10_ EID_50_/mL (A/South Africa/3626/2013). To normalize this disparity, the optimal titer value was given the nominal value of 100%. For each virus, the titer at 40 °C was then calculated as a percentage relative to this nominal value, as described in the Materials and Methods Section.

**Figure 2:**
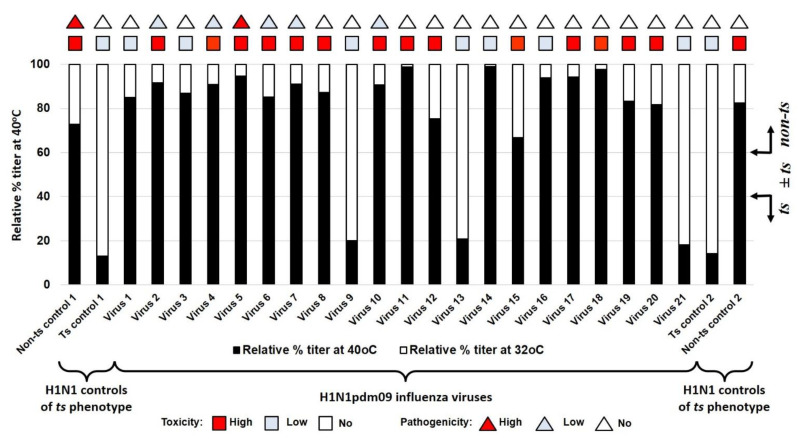
Sensitivity of reproduction of A(H1N1)pdm09 influenza viruses to the elevated temperature of 40 °C. Black bars—relative percentage titer at 40 °C compare to infectivity at 32 °C. triangles—pathogenicity for mice. Square—toxicity for mice. *Non–ts* control 1—A/PR/8/1934; *ts* control 1—A/Florida/3/2006; virus 1—A/California/07/2009; virus 2—A/Bolivia/559/2013; virus 3—A/Mississippi/10/2013; virus 4—A/New Hampshire/04/2013; virus 5—A/South Africa/3626/2013; virus 6—A/Florida/62/2014; virus 7—A/Laos/1187/2014; virus 8—A/New York/61/2015; virus 9—A/Slovenia/2903/2015; virus 10—A/Bangladesh/3002/2015; virus 11—A/Newcastle/67/2017; virus 12—A/South Australia/272/2017; virus 13—A/New Jersey/13/2018; virus 14—A/Darwin/123/2018; virus 15—A/Brisbane/02/2018; virus 16—A/lowa/59/2018; virus 17—A/lowa/12/2019; virus 18—A/Victoria/2570/2019; virus 19—A/Guangdong-Maonan/SWL1536/2019; virus 20—A/Arkansas/08/2020; virus 21—A/Indiana/02/2020; *ts* control 2—A/Solomon Islands/3/2006; *non–ts* control 2—A/New Caledonia/20/1999. The red square indicates a strain of high toxicity. The red triangle indicates strain of high pathogenicity. The pale blue square indicates a strain of low or no toxicity. The pale blue triangle indicates strain of low or no pathogenicity.

Of the 21 studied viruses, none fell in the intermediate phase and they all had a clear phenotype; only three viruses had a *ts* phenotype (9, A/Slovenia/2903/2015; 13, A/New Jersey/13/2018; 21, A/Indiana/02/2020), whereas the rest of them had a *non-ts* phenotype. The studied virus kept a *non–ts* phenotype from 2009 until 2015 when the first *ts* strain (9, A/Slovenia/2903/2015) appeared, and after that, the phenotype fluctuated between *ts* and *non-ts*. The *ts* phenotype appeared in two other strains (13, A/New Jersey/13/2018; 21, A/Indiana/02/2020) in 2018 and 2020, respectively (Figure 2).

### 3.4. Thermal Stability of the Hemagglutinin of A(H1N1)pdm09 Viruses

In this study, nine of the studied viruses (virus 5, A/South Africa/3626/2013; virus 6, A/Florida/62/2014; virus 7, A/Laos/1187/2014; virus 8, A/New York/61/2015; virus 10, A/Bangladesh/3002/2015; virus 16, A/lowa/59/2018; virus 17, A/lowa/12/2019; virus 19, A/Guangdong-Maonan/SWL1536/2019; virus 20, A/Arkansas/08/2020) had a highly stable HA, while 12 of them (virus 1, A/California/07/2009; virus 2, A/Bolivia/559/2013; virus 3, A/Mississippi/10/2013; virus 4, A/New Hampshire/04/2013; virus 9, A/Slovenia/2903/2015; virus 11, A/Newcastle/67/2017; virus 12, A/South Australia/272/2017; virus 13, A/New Jersey/13/2018; virus 14, A/Darwin/123/2018; virus 15, A/Brisbane/02/2018; virus 18, A/Victoria/2570/2019; virus 21, A/Indiana/02/2020) had low thermal stability of HA (Table 4). The HA of the A/South Africa/3626/2013 virus recorded the highest activity temperature threshold, losing its activity at 65 °C (Table 4).

**Figure 3:**
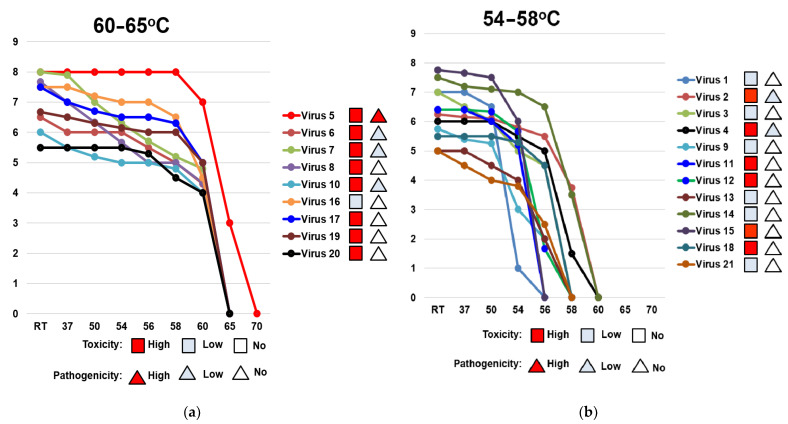
Comparison of the dynamic of losses of thermal stability of the hemagglutinin of A(H1N1)pdm09 viruses circulating in 2009–2020. HA activity temperature threshold is (**a**) 60–65 °C (high thermal stability); (**b**) 54–58 °C (low thermal stability). RT—room temperature. X-axis—temperature (°C). Y-axis—log2 HA titer. Virus 1—A/California/07/2009; virus 2—A/Bolivia/559/2013; virus 3—A/Mississippi/10/2013; virus 4—A/New Hampshire/04/2013; virus 5—A/South Africa/3626/2013; virus 6—A/Florida/62/2014; virus 7—A/Laos/1187/2014; virus 8—A/New York/61/2015; virus 9—A/Slovenia/2903/2015; virus 10—A/Bangladesh/3002/2015; virus 11—A/Newcastle/67/2017; virus 12—A/South Australia/272/2017; virus 13—A/New Jersey/13/2018; virus 14—A/Darwin/123/2018; virus 15—A/Brisbane/02/2018; virus 16—A/lowa/59/2018; virus 17—A/lowa/12/2019; virus 18—A/Victoria/2570/2019; virus 19—A/Guangdong-Maonan/SWL1536/2019; virus 20—A/Arkansas/08/2020; virus 21—A/Indiana/02/2020. The red square indicates a strain of high toxicity. The red triangle indicates strain of high pathogenicity. The pale blue square indicates a strain of low or no toxicity. The pale blue triangle indicates strain of low or no pathogenicity.

The dynamics of how losses of HA activity are affected by increasing the temperature from the room temperature (25 °C) up to 70 °C are shown in Figure 3. The studied viruses were separated into two groups depending on their HA activity temperature threshold. The first group had high HA stability (HA activity temperature threshold 60–65 °C; Figure 3a) and the second group had low HA stability (HA activity temperature threshold 54–58 °C; (Figure 3b).

### 3.5. Phylogenetic Trees of A(H1N1)pdm09 Influenza Viruses Genes

**Figure 4:**
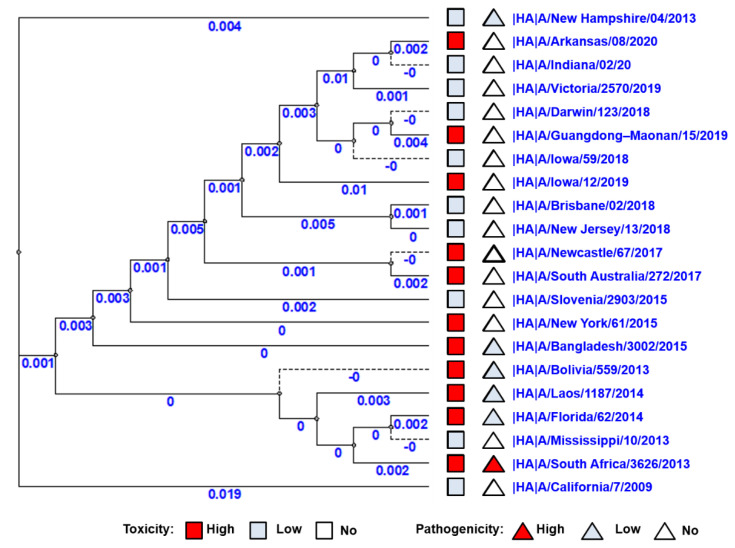
Phylogenetic tree of HA genes of A(H1N1)pdm09 influenza viruses used in this study. The red square indicates a strain of high toxicity. The red triangle indicates a strain of high pathogenicity. The pale blue square indicates a strain of low or no toxicity. The pale blue triangle indicates a strain of low or no pathogenicity.

Phylogenetic trees of all 10 viral proteins were built to study the possible correlation between the viral toxic and pathological properties and their gene sequence. No correlation was observed between virus distributions on the phylogenetic tree of HA genes and toxic/pathological properties (Figure 4). Thus, to identify the possible role of other genes (NA, PA, PB1, PB2, M1, M2, NP, NEP, NS1) in the manifestation of pathogenicity and toxicity, phylogenetic trees for the remaining nine viral proteins were constructed; again, no clear correlation was detected (Supplemental Figures S1–S9).

### 3.6. Presumed Molecular Basis for High Pathogenicity and Toxicity as A(H1N1)pdm09 Viral Properties

Since the distribution of viruses on phylogenetic trees did not provide clear explanations for viral pathogenicity and toxicity, we sought more detailed information at the molecular level. Amino acid sequence alignment was performed for all viral protein sequences of the 21 studied viruses. We found no one mutation or amino acid substitution in all eight genes, coding for both HA and NA and internal proteins, which could explain the different levels of toxicity of the studied viruses.

**Figure 5:**
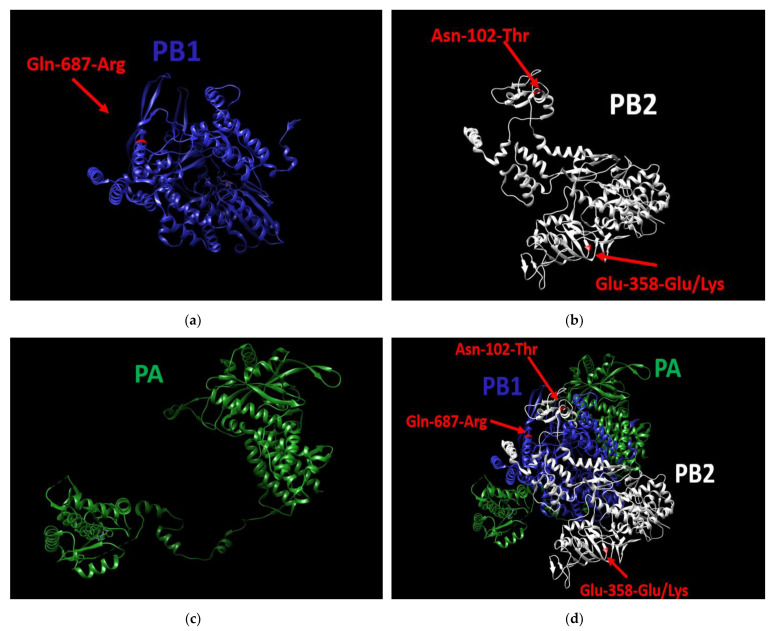
3D structure of the polymerase complex of A/South Africa/3626/2013 virus. (**a**) the PB1 protein contains the unique substitution (Gln-687-Arg), (**b**): the PB2 protein contains two unique substitutions (Asn-102-Thr, Glu-358-Glu/Lys), (**c**) the PA protein does not contain unique mutations, (**d**) Structure of the entire polymerase complex with the three substitutions. UCSF Chimera 1.15, was used to build the 3D structure.

As for pathogenicity, in the previous step of the study we demonstrated that of the 21 tested viruses, only A/South Africa/3626/2013 demonstrated a high pathogenicity level (see Section “Pathogenicity for Mice”). It was shown that A/South Africa/3626/2013 used in our experiments had no unique mutations in HA, NA, M1, M2, NP, NEP, or NS1. On the other hand, in two out of three genes coding for the polymerase complex, three unique substitutions were found (Figure 5d); specifically, these were the Asn-102-Thr substitution in PB2, Glu-358-Glu/Lys heterogeneity substitution also in PB2 (Figure 5b), and Gln-687-Arg substitution in PB1 (Figure 5a). The PA gene does not contain unique substitutions (Figure 5c).

**Figure 6:**
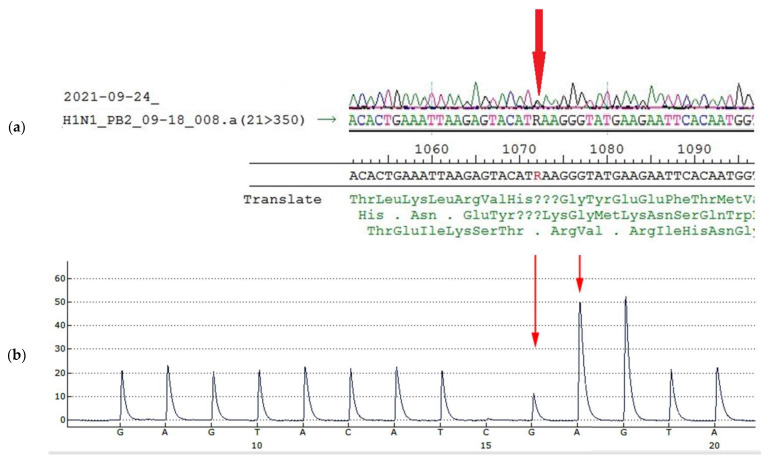
Sanger sequencing and pyrosequencing of A/South Africa/3626/2013 virus used for mice infection (egg passage E4) (**a**): Sanger sequencing of PB2 gene fragment, (**b**): Detection of the nucleotide sequence in PB2 fragment 1064–1079 of A/South Africa/3626/2013 virus used for mice infection (egg passage E4) by pyrosequencing. The peak highness in G position (**b**, long arrow) is half-height, peak A (**b**, short arrow) is less than 3 height, (in case of AAA sequence the peak would be higher than 3, because of slightly higher fluorescent of A due to protocol peculiarities). The approximate ratio of G/A components in the 1072 position is equal.

Glu-358-Glu/Lys heterogeneity in PB2 results from heterogeneity A/G in the 1072 nucleotide position of the PB2 gene segment (Figure 6a, Table 5). To detect the ratio of each component in the population, pyrosequencing of this position was performed. According to pyrosequencing reaction results, the ratio of variants was about 1: (Figure 6b, Table 5).

**Figure 7:**
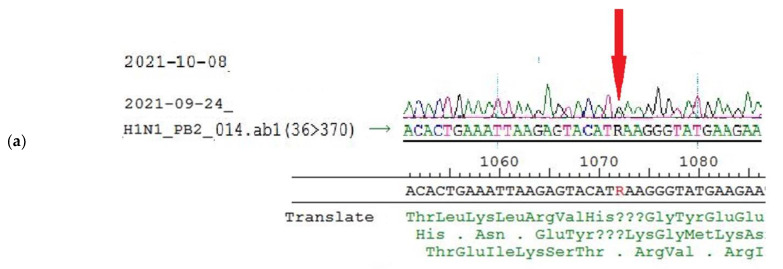

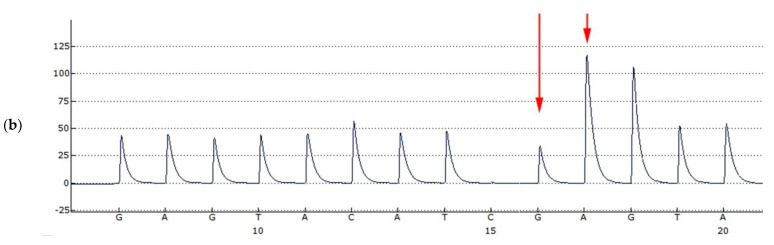
Sanger sequencing and pyrosequencing of A/South Africa/3626/2013 virus of mice isolates (**a**): Sanger sequencing of PB2 gene fragment, (**b**): Detection of the nucleotide sequence in PB2 fragment 1064–1079 by pyrosequencing. Arrows show the peaks of interest.

Further investigations into the heterogeneity A/G in 1072 nucleotide position in PB2 gene segment were conducted, including sequencing of viral RNA extracted from mice lung tissue on the third day after the infection (E4 + one passage in mice). Sanger sequencing and pyrosequencing of virus population isolated from mice lungs revealed the persistence of 1072 A/G heterogeneity (Figure 7a,b).

Interestingly, the sequence of the PB2 segment for the A/South Africa/3626/2013 virus E1E2/E3 passage (GISAID isolate ID EPI_ISL_175880) also contained 1072 A/G heterogeneity (Table 5). In the light of previous results, the alignment study was expanded to include an additional 134 influenza A (H1N1)pdm09 viruses isolated from 2009 to 2021 and available on the GISAID platform (the GISAID isolates’ IDs are listed in Supplemental Table S1), where the three abovementioned substitutions remained unique for A/South Africa/3626/2013 (Table 5).

## 4. Discussion

The primary objective of this work was to study the evolutionary variability of certain biological traits of influenza A(H1N1)pdm09 virus strains that have been circulating since 2009 in order to determine how their biological properties have been changing during their circulation. We analyzed 21 A(H1N1)pdm09 viruses—starting from A/California/07/2009 and ending with last year’s strains—according to a variety of parameters, in particular, the pathogenicity and toxicity for mice, the temperature sensitivity of their reproduction in developing chicken embryos, and the thermal stability of hemagglutinin. We sought to detect any relationship between these traits and possible mutational changes.

*Non-ts* phenotype viruses can infect lower respiratory tracts as they can replicate at temperatures higher than 37 °C, which increases their pathogenicity and allows them to resist the organism’s response [35,36]. For this reason, temperature-sensitive *(ts*) viruses are much less virulent [35]. Temperature sensitivity is also an essential property required for the development of attenuated reassortant strains for live influenza vaccines [37,38]. It has been suggested that the circulation of only temperature-sensitive viruses for several years can be considered as a sign of the coming appearance of a new virus that is antigenically distant from the circulating strains [39].

This suggestion is supported by the observation that the evolutionary variability of the *ts/non-ts* phenotype has a fluctuating trend where the *non-ts* phenotype is dominant at the beginning of the pandemic cycle. Subsequently, the percentage of *ts* viruses after the emergence of a new strain increases stably over the years [14,39]. For instance, in 1949–1957, *ts* isolates accounted for 8.3% of all investigated A(H1N1) viruses, whereas they made up 76.5% in 1977–1978 [35], which provided the opportunity for new *non-ts* strains (A/Khabarovsk/90/77 “Russian flu”) to appear and circulate, causing the 1977 pandemic [14].

The same phenomenon was observed just before the 2009 influenza pandemic. All influenza A (H1N1), influenza A (H3N2), and influenza B viruses isolated from 2006 to 2009 had a *ts* phenotype [14]. In other words, the *ts* viruses circulated until 2009, when they were replaced by the viciously spreading *non-ts* A/California/07/09 (H1N1)pdm influenza virus, causing the 2009 pandemic. The A/California/07/09 (H1N1)pdm virus has begun another wave of circulation of *non-ts* viruses.

Although there were only 21 viruses in our study group, the fluctuating trend persevered, whereby drift variants of the A/California/07/09 (H1N1)pdm virus kept the *non-ts* phenotype until 2015, after which the first *ts* strain (9, A/Slovenia/2903/2015) appeared and the proportion of the *ts* phenotype started to increase gradually, indicating that the *ts* phenotype will dominate in the coming years.

The newly emerged human influenza A viruses might have kept two of the three genes, as a minimum, of the polymerase complex from their progenitor, which could explain the ability of these viruses to withstand and produce new copies in temperatures over 37 °C since these progenitors used to replicate in animals that have a higher physiological body temperature than humans, such as birds or pigs [14]. In addition, a variety of studies showed that the *ts/non-ts* phenotype of influenza viruses is connected with mutations in the protein PB2 [40,41,42].

The phenomenon of influenza viral toxicity has been known since the 1940s [16,43]. However, the mechanism of viral toxicity is still poorly studied. Most works were published in the 1990s [44,45,46]. The idea was that the toxicity of the influenza virus is probably caused by viral dsRNA, produced during viral replication, either directly because it shares many biological and physical properties of bacterial toxins or as a result of the interferon (IFN) it induces [46,47]. The direct mechanism theory is supported by the antitoxic effect of rimantadine, which works as an M2 ion channel membrane protein blocker, preventing detachment of the viral RNP from the matrix, which blocks influenza A virus entry into the cells [37,38]. However, rimantadine did not show specific activity against influenza B viruses’ primary pneumonia [48]. Furthermore, though rimantadine created a significant reduction in the mortality of mice from toxic effects caused by influenza A viruses, influenza B viruses, or exotoxin of *Staphylococcus aureus*, it did not prevent the toxic effect of adrenaline [48]. This suggests that bacterial toxins and influenza viruses might have the same toxic mechanism that can be eliminated by rimantadine even if the strain is resistant to it [48]. Moreover, viral dsRNA causes cytotoxic effects through two mechanisms: either it inhibits protein synthesis indirectly through its induction of 2’–5’ oligo (A) synthetase and/or a dsRNA-dependent protein kinase, or it enhances degradation of ssRNA through activation of the endonuclease RNase. These cellular effects may cause local tissue necrosis in addition to the classical antiviral effect of IFN. During cell lysis, dsRNA, which can resist degradation by intracellular nucleases [47], is released into the extracellular environment and interacts with macrophages, invoking cytokine production [46,47] and triggering the toxic effect [44,45]. These toxicity mechanisms might explain why there is no one mutation or amino acid substitution that can be associated with the different levels of toxicity of the studied viruses.

**Figure 8:**
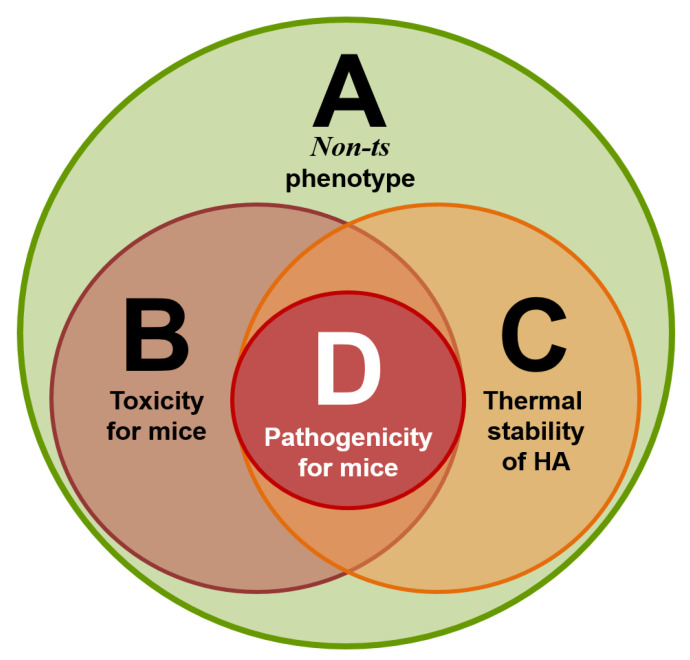
Four circle diagram: correlations of four sets of virus properties—A (*non-ts* phenotype), B (toxicity for mice), C (thermal stability of the hemagglutinin), and D (pathogenicity for mice).

Our research concluded that for a virus to have properties such as toxicity, or the thermal stability of the hemagglutinin, it is necessary but not sufficient to be characterized as a *non-ts* phenotype. In other words, if the virus has a toxic effect or thermally stable hemagglutinin, it is adequate evidence that the virus has a *non-ts* phenotype but it is not the other way around. Furthermore, having a *non-ts* phenotype, toxicity and thermally stable hemagglutinin are necessary but not sufficient for the virus to become pathogenic for laboratory animals, indicating that there are still some properties that might also contribute to the degree of pathogenicity. The strongest candidates are the virus transmissibility and resistance to sera non-specific inhibitors. However, if the virus is pathogenic for laboratory animals, that means that it has all of the other three properties (Figure 8).

Earlier, we showed that the A/South Africa/3626/2013 influenza virus has the unique property of high pathogenicity for mice without preliminary adaptation to them, but the ancestor virus A/California/07/2009 does not have such a property [29]. The interaction between the influenza virus and the host cells on the molecular level defined the viral virulence and its ability to infect this particular host. In addition, the virus has different methods of avoiding the antiviral response of the host. The process of the adaptation of the influenza virus to a new host requires mutations and/or the reassortment of the viral genome, and these events present themselves as a level of pathogenicity [49].

Practically all of the viral genes participate in virus pathogenicity; this fact was established in numerous studies [50,51,52,53,54,55,56,57,58,59,60,61]. HA and NA always work in harmony, are responsible for tissue tropism and interspecies transmission [49], and strongly affect transmissibility between humans [62], while NS1 virus proteins increase the viral pathogenicity by working as interferon antagonists and inhibiting the interferon transcription factors [63], which give the virus the ability to escape from the immune system. The polymerase complex also plays an essential role in viral pathogenicity. For instance, to adapt to mammals, one of the avian influenza viral strategies is increasing the activity of the viral polymerase complex [10]. Moreover, the polymerase complex plays a special role in the pathogenicity or attenuation of influenza viruses. Attenuation may be considered as the reverse of pathogenicity [18]. In particular, mutations in PB2 and PB1 can convert the *non-ts* phenotype to *ts*, resulting in the attenuation of the strain. For instance, the modification of a cold-adapted attenuated influenza virus model and its wild-type (WT) progenitor using different methods (classical reassortment or plasmid-based reverse genetics system) demonstrated how replacing PB2 mutant genes in the attenuated model with PB2 from its WT progenitor results in a *non-ts* reassortant. On the other hand, replacing PB2 genes in the WT progenitor with mutant PB2 from the attenuated model results in a *ts* attenuated reassortant. The same results were obtained with a PB1 substitution [22,23,24,25].

Our molecular study showed that the A/South Africa/3626/2013 virus has three unique mutations in the polymerase complex (two in PB2 Asn-102-Thr, Glu-358-Glu/Lys and one in PB1 Gln-687-Arg). Gln-687-Arg localized in the C-terminus of PB1 [64]. Residues 678–757 in the C-terminus of PB1 form a binding site, which is required for tight-binding with PB2 through its N-terminus. Mutations in this position strongly affect polymerase activity [65]. Asn-102-Thr falls in the (PB2-N1) subdomain of the N-terminal third, and this subdomain acts as a support for the PB1 thumb domain [64]. Due to these data, we can assume that the two mutations might increase the polymerase stability and thus its activity, which could explain the high pathogenicity of the A/South Africa/3626/2013 virus.

Regarding the Glu-358-Glu/Lys substitution in PB2, Glu in this position is highly conservative among different influenza subtypes (in the alignment of 517 PB2 protein sequences of H1N1, H3N2, H2N2, H5N1, H5N8, H7N7, H7N9, and H9N2 animal and human influenza isolates; see Table 4). The Lys variant was detected in only one A/northern shoveler/Mississippi/11OS202/2011 (H7N7) influenza virus (GISAID sequence ID EPI419570) [34]. This amino acid position is part of the cap-binding domain of the PB2 protein [64], which suggests that this mutation might directly affect the polymerase activity. However, in our study, the heterogeneity persisted with an equal ratio even after passage in mice, demonstrating that the two variants had been replicated equally in mice’s lungs, which indicates that this substitution might not sufficiently affect the viral pathogenicity. Nevertheless, further investigation—including the reassortment of the gene fragments containing these three mutations—is required to affirm the roles of these three mutations in viral pathogenicity.

## 5. Conclusions

For the first time, a comparative analysis of modern A(H1N1)pdm09 influenza viruses, according to their essential biological characteristics and how are they associated with changes at the molecular level, was conducted. We know that viral pathogenicity results from the contributions of many properties. In this study, we focused on the roles of the non-ts phenotype, toxicity, and HA thermal stability in the manifestation of pathogenicity. For high pathogenicity, the virus must have all three properties. Each of these properties is indispensable and plays a key role in viral pathogenicity in a mice model.

An alignment analysis was carried out to answer the question of what the causes are of high or low pathogenicity of influenza viruses A(H1N1)pdm09 at the molecular level. It was previously shown that genes coding for the polymerase complex play a key role in the influenza A virus pathogenicity. In this study, three unique mutations were found in the polymerase complex of the A/South Africa/3626/2013 influenza virus that might contribute to its function, thus explaining the high pathogenicity of this virus.

## 6. Limitations

In the genome analysis of the study the sample size included 154 influenza A H1N1 viruses from which only 21 reference viruses were available for the authors to perform a comparative analysis of the main biological characteristics of the viruses.

## Figures and Tables

**Table 1:** A list of influenza A(H1N1)pdm09 viruses used in this study.

A(H1N1)pdm09 Strain No/Designation		Source	Clade/Subclade
^1^ 1	A/CALIFORNIA/07/2009	^2^ CDC ID 2009712112	^6^ n.a.
2	A/Bolivia/559/2013	CDC ID 2013760341	6B
3	A/Mississippi/10/2013	CDC ID 2014700252	6B
4	A/New Hampshire/04/2013	CDC ID 2013845913	6B
5	A/South Africa/3626/2013	CDC ID 2014701384	6B
6	A/Florida/62/2014	CDC ID 3000097732	6B
7	A/Laos/1187/2014	CDC ID 3000095267	6B.1
8	A/New York/61/2015	CDC ID 3000412528	6B.1
9	A/Slovenia/2903/2015	^3^ NIBSC lot 41730	6B.1
10	A/Bangladesh/3002/2015	CDC ID 3000411636	6B
11	A/Newcastle/67/2017	WHO-CC ID 322513509	6B.1
12	A/South Australia/272/2017	WHO-CC ID 17-62350735	6B.1A
13	A/New Jersey/13/2018	CDC ID 3026016623	6B.1A.1
14	A/Darwin/123/2018	WHO-CC ID 10010145	6B.1A5A
15	A/Brisbane/02/2018	^4^ WHO-CC ID SS18S304	6B.1A.1
16	A/lowa/59/2018	CDC ID 3026018252	6B.1A5A
17	A/lowa/12/2019	CDC ID 3026019744	6B.1A5B
18	A/Victoria/2570/2019	WHO-CC ID 10033413	6B.1A5A-156K
19	A/Guangdong-Maonan/SWL1536/2019	NIBSC code 19/294	6B.1A5A-187A
20	A/Indiana/02/2020	CDC ID 3026055186	6B.1A5A-156K
21	A/Arkansas/08/2020	CDC ID 3026055197	6B.1A5A-156K
A(H1N1) past influenza viruses used as controls for evaluation of *ts/non-ts* phenotype			
22	*non-ts* control 1, A/PR/8/34	^5^ ATCC ID VR-1469	^6^ n.a.
23	*non-ts* control 2, A/New Caledonia/20/99	NIBSC code 07/226	^7^ delta-like clade
24	*ts* control 1, A/Florida/3/06	ATCC ID VR-1893	^7^ delta2
25	*ts* control 2, A/Solomon Islands/3/06	NIBSC code 06/236	^7^ delta-like clade

^1^ Viruses were numbered from 1 to 21 and will be referred to throughout the article using the assigned numbers. ^2^ CDC: Centers for Disease Control and Prevention, Atlanta, GA, the US. ^3^ NIBSC: the National Institute for Biological Standards and Control, Hertfordshire, UK. ^4^ WHO-CC: World Health Organization Collaborating Centre for Reference & Research on Influenza, Melbourne, Australia. ^5^ ATCC: American Type Culture Collection, Manassas, VA, the US. ^6^ n.a.: not applicable. ^7^ According to [26].

**Table 2:** Assessment of the level of manifestation of signs of toxicity and pathogenicity in mice.

Parameters	High	Low	No (Absence)
Toxicity for mice, %, on day six	>50 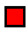	≤50 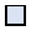	0 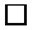
Pathogenicity for mice, on day 14, log_10_ LD_50_	≥4.0 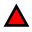	≤4.0 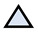	<1.5 ^1^ 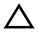

^1^ The threshold limit value.

**Table 3:** The main characteristics of 21 A(H1N1)pdm09 influenza viruses tested in vivo.

A(H1N1)pdm09 Virus No/Designation		Acute Toxicity on D6 ^1^		Pathogenicity on Day 14 ^2^		
		Lethality, %	Level of Toxicity	log LD_50_^3^	1 LD_50_ in log EID_50_/mL ^4^	Level of Pathogenicity
1	A/CALIFORNIA/7/2009	10%	low	<1.5	>8.0	No
2	A/Bolivia/559/2013	100%	high	1.6	6.6	low
3	A/Mississippi/10/2013	40%	low	<1.5	>8.4	No
4	A/New Hampshire/04/2013	50%	low	1.7	6.5	low
5	A/South Africa/3626/2013	80%	high	5.0	4.2	high
6	A/Florida/62/2014	80%	high	2.0	5.5	low
7	A/Laos/1187/2014	100%	high	1.9	6.1	low
8	A/New York/61/2015	60%	high	<1.5	6.6	No
9	A/Slovenia/2903/2015	20%	low	<1.5	>8.1	No
10	A/Bangladesh/3002/2015	100%	high	1.6	6.6	low
11	A/Newcastle/67/2017	80%	high	<1.5	>8.4	No
12	A/South Australia/272/2017	90%	high	<1.5	>8.4	No
13	A/New Jersey/13/2018	10%	low	<1.5	>7.2	No
14	A/Darwin/123/2018	27%	low	<1.5	>9.0	No
15	A/Brisbane/02/2018	50%	low	<1.5	>8.5	No
16	A/Iowa/59/2018	40%	low	<1.5	>8.2	No
17	A/Iowa/12/2019	100%	high	<1.5	6.6	No
18	A/Victoria/2570/2019	50%	low	<1.5	>8.4	No
19	A/Guangdong-Maonan/SWL1536/2019	90%	high	<1.5	>9.0	No
20	A/Arkansas/08/2020	80%	high	<1.5	>8.2	No
21	A/Indiana/02/20	20%	low	<1.5	>8.3	No

^1^ Lethality on Day six of the experiment, % (for the study of viral acute toxicity). ^2^ Lethality on Day 14 of the experiment, % (for the study of viral pneumonia).^3^ Threshhold limit. ^4^ Expressed as the log_10_ EID_50_/mL required to give 1 LD_50_.

**Table 4:** The lowest temperature destroying the hemagglutinin activity.

A(H1N1)pdm09 Virus No/Designation		HA Activity Temp. Threshold ^1^	
1	A/CALIFORNIA/07/2009	54 °C	Low
2	A/Bolivia/559/2013	58 °C	Low
3	A/Mississippi/10/2013	56 °C	Low
4	A/New Hampshire/04/2013	58 °C	Low
5	A/South Africa/3626/2013	65 °C	High
6	A/Florida/62/2014	60 °C	High
7	A/Laos/1187/2014	60 °C	High
8	A/New York/61/2015	60 °C	High
9	A/Slovenia/2903/2015	56 °C	Low
10	A/Bangladesh/3002/2015	60 °C	High
11	A/Newcastle/67/2017	54 °C	Low
12	A/South Australia/272/2017	58 °C	Low
13	A/New Jersey/13/2018	56 °C	Low
14	A/Darwin/123/2018	58 °C	Low
15	A/Brisbane/02/2018	54 °C	Low
16	A/lowa/59/2018	60 °C	High
17	A/lowa/12/2019	60 °C	High
18	A/Victoria/2570/2019	56 °C	Low
19	A/Guangdong-Maonan/SWL1536/2019	60 °C	High
20	A/Arkansas/08/2020	60 °C	High
21	A/Indiana/02/2020	56 °C	Low

^1^ The lowest temperature destroys the HA activity.

**Table 5:** Unique substitutions in amino acid sequence of A/South Africa/3626/2013 (H1N1)pdm09 influenza virus proteins.

Sequence Resource	Gene Segment	Nt Position	Nt Substitution	RNA Sequence	AA Position	AA Substitution
GISAID isolate (ID EPI_ISL_175880)	PB1	2060	A 2060 G	C**G**G	687	Gln-687-Arg
	PB2	305	A 305 C	A**C**T	102	Asn-102-Thr
		1071	G 1071 R	**R**AA ^1^	358	Glu-358-Glu/Lys
Virus stock used in this study	PB2	1071	G 1071 R	**R**AA ^1^	358	Glu-358-Glu/Lys
Virus isolates from mice	PB2	1071	G 1071 R	**R**AA ^1^	358	Glu-358-Glu/Lys
154 ^3^ A(H1N1)pdm09 isolates from the GISAID database	PB1	2060	A ^2^	C**A**G	687	Gln ^2^
	PB2	305	A ^2^	A**A**T	102	Asn ^2^
		1071	G ^2^	**G**AA	358	Glu ^2^
517 H1N1, H3N2, H2N2, H5N1, H5N8, H7N7, H7N9, H9N2 isolates from the GISAID database [34]	PB2	1071	G ^2^	**G**AA	358	Glu ^2^

^1^ R is a purine (G or A). ^2^ No substitution was detected in this position. ^3^ 134 isolates from the GISAID database in addition to the 20 viruses tested in this study.

## Data Availability

All relevant data are within the manuscript.

## Supplementary Materials

The following are available online at https://www.mdpi.com/article/10.3390/vaccines10030395/s1, Figure S1: Phylogenetic tree of NA genes of A(H1N1)pdm09 influenza viruses used in this study, Figure S2: Phylogenetic tree of PA genes of A(H1N1)pdm09 influenza viruses used in this study, Figure S3: Phylogenetic tree of PB1 genes of A(H1N1)pdm09 influenza viruses used in this study, Figure S4: Phylogenetic tree of PB2 genes of A(H1N1)pdm09 influenza viruses used in this study, Figure S5: Phylogenetic tree of M1 genes of A(H1N1)pdm09 influenza viruses used in this study, Figure S6: Phylogenetic tree of M2 genes of A(H1N1)pdm09 influenza viruses used in this study, Figure S7: Phylogenetic tree of NP genes of A(H1N1)pdm09 influenza viruses used in this study, Figure S8: Phylogenetic tree of NEP genes of A(H1N1)pdm09 influenza viruses used in this study, Figure S9: Phylogenetic tree of NS1 genes of A(H1N1)pdm09 influenza viruses used in this study, Table S1: IDs of 150 available in GISAID sequences of A(H1N1)pdm09 influenza viruses.
