# Supplementary figures and images for: Understanding the Variability of Certain Biological Properties of H1N1pdm09 Influenza Viruses
Source: Vaccines (Basel). 2022 Mar 3;10(3):395. doi: 10.3390/vaccines10030395 (PMC8954537; doi:10.3390/vaccines10030395)

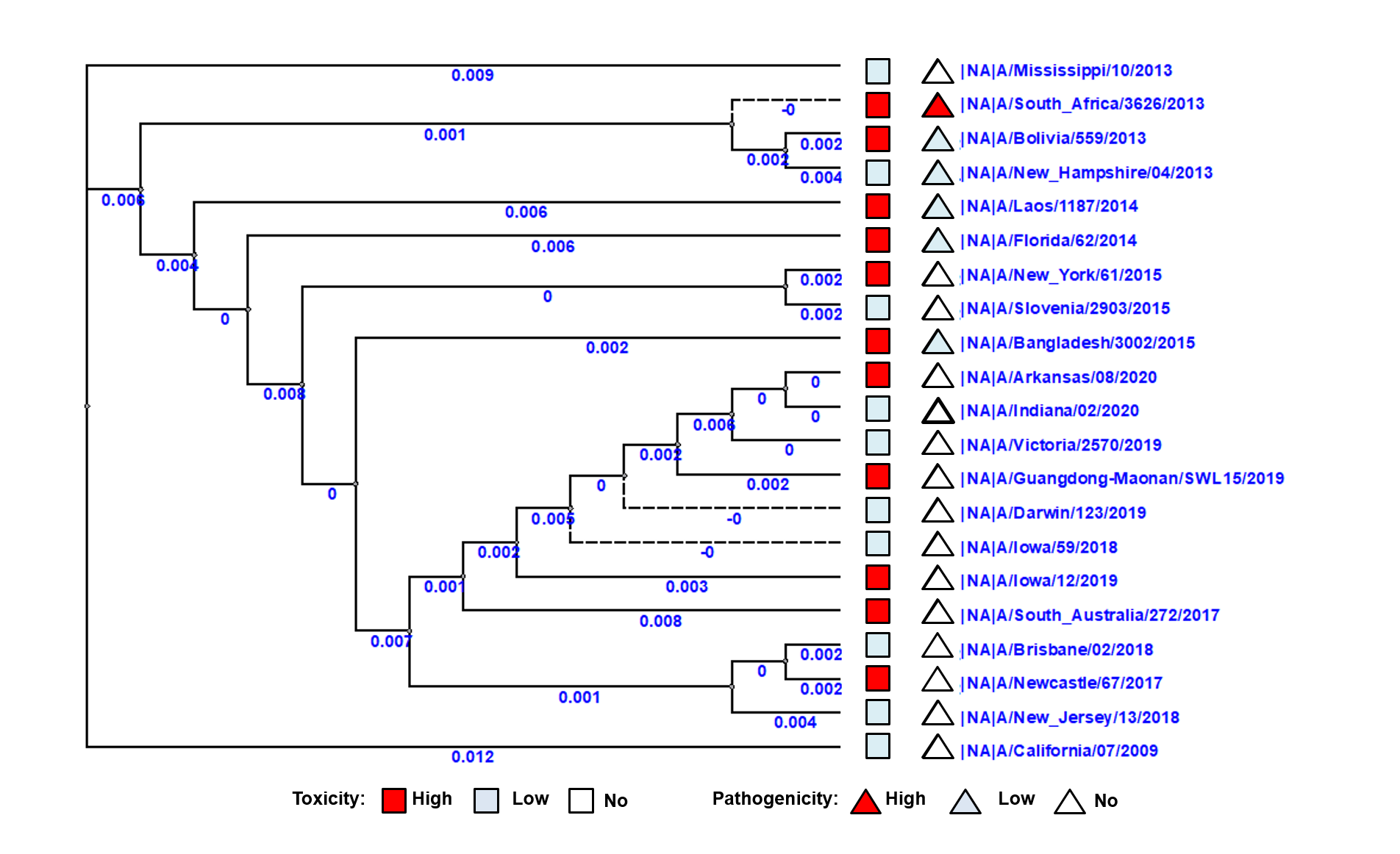

Supplement: Supplementary file 1 [file vaccines-10-00395-s001.zip › vaccines-1522373-supplementary/Figure S1.tif]

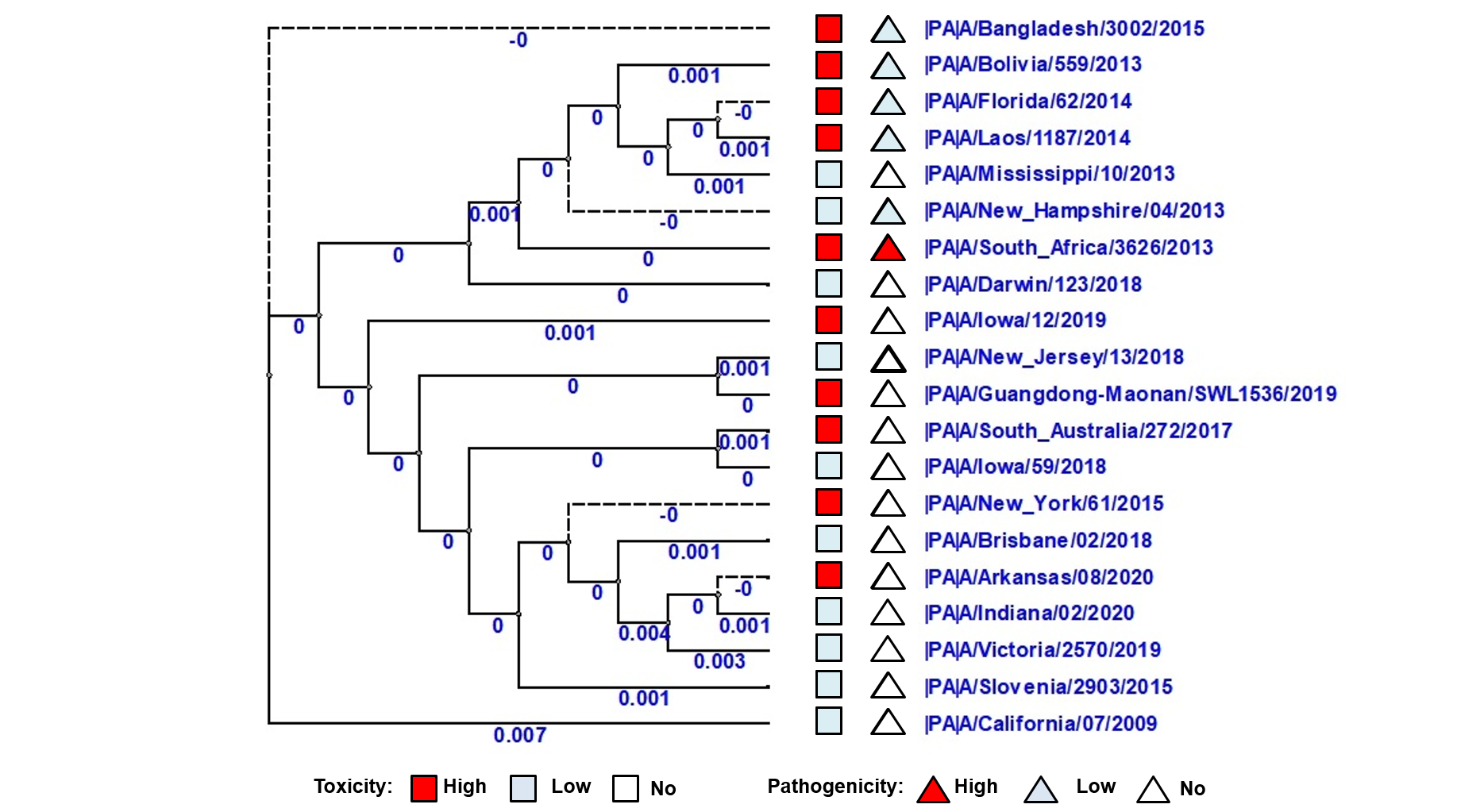

Supplement: Supplementary file 1 [file vaccines-10-00395-s001.zip › vaccines-1522373-supplementary/Figure S2.tif]

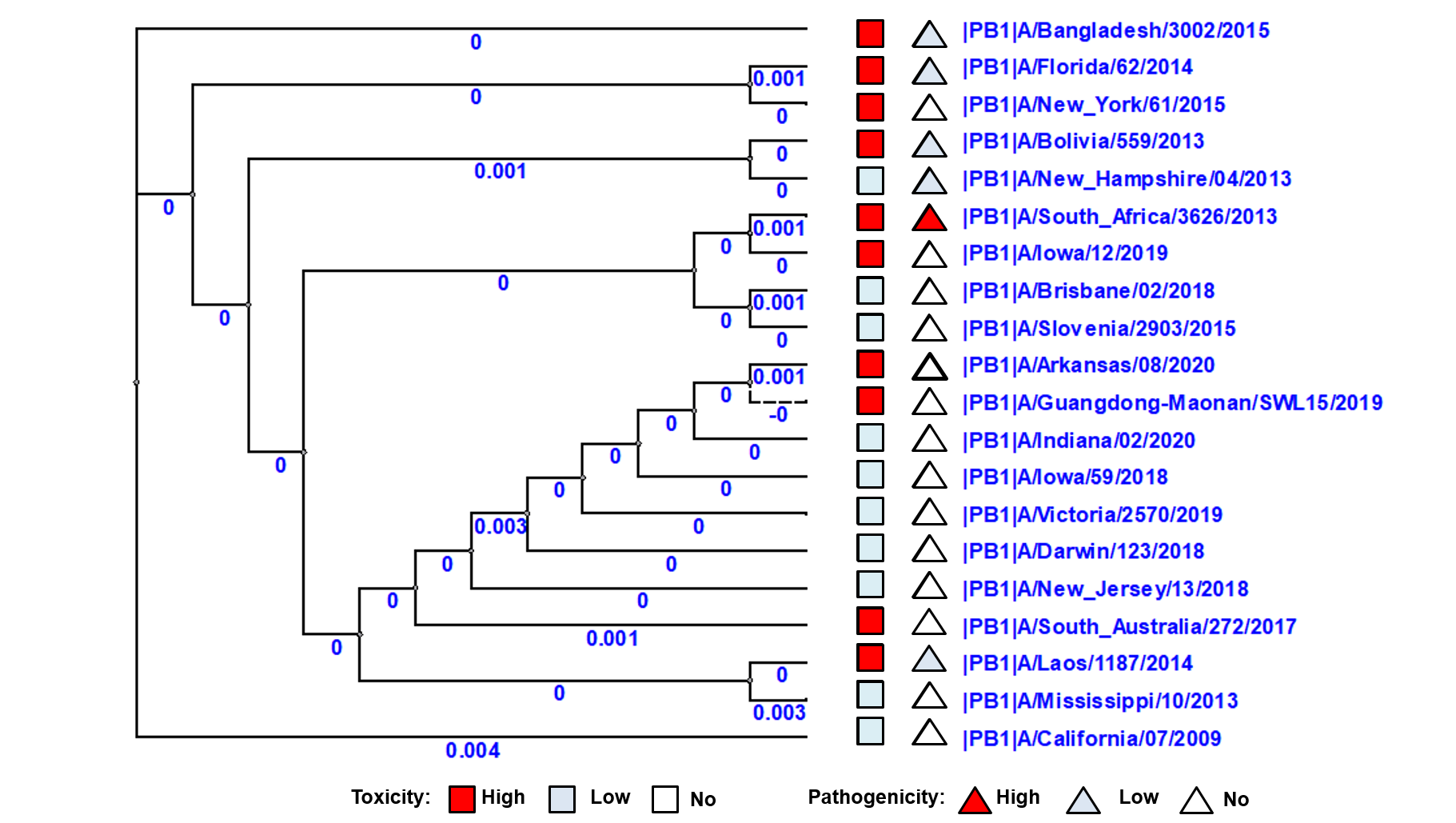

Supplement: Supplementary file 1 [file vaccines-10-00395-s001.zip › vaccines-1522373-supplementary/Figure S3.tif]

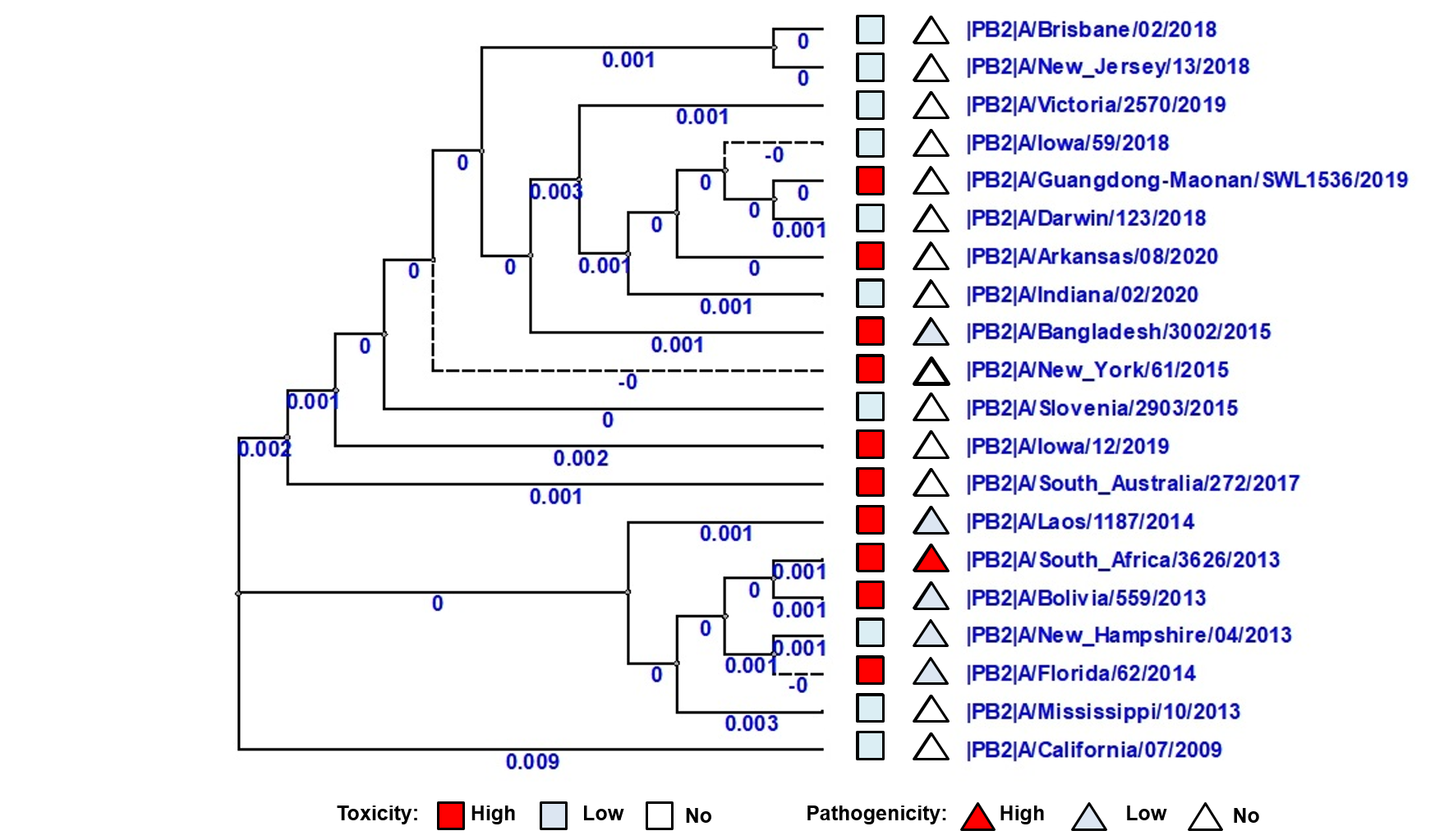

Supplement: Supplementary file 1 [file vaccines-10-00395-s001.zip › vaccines-1522373-supplementary/Figure S4.tif]

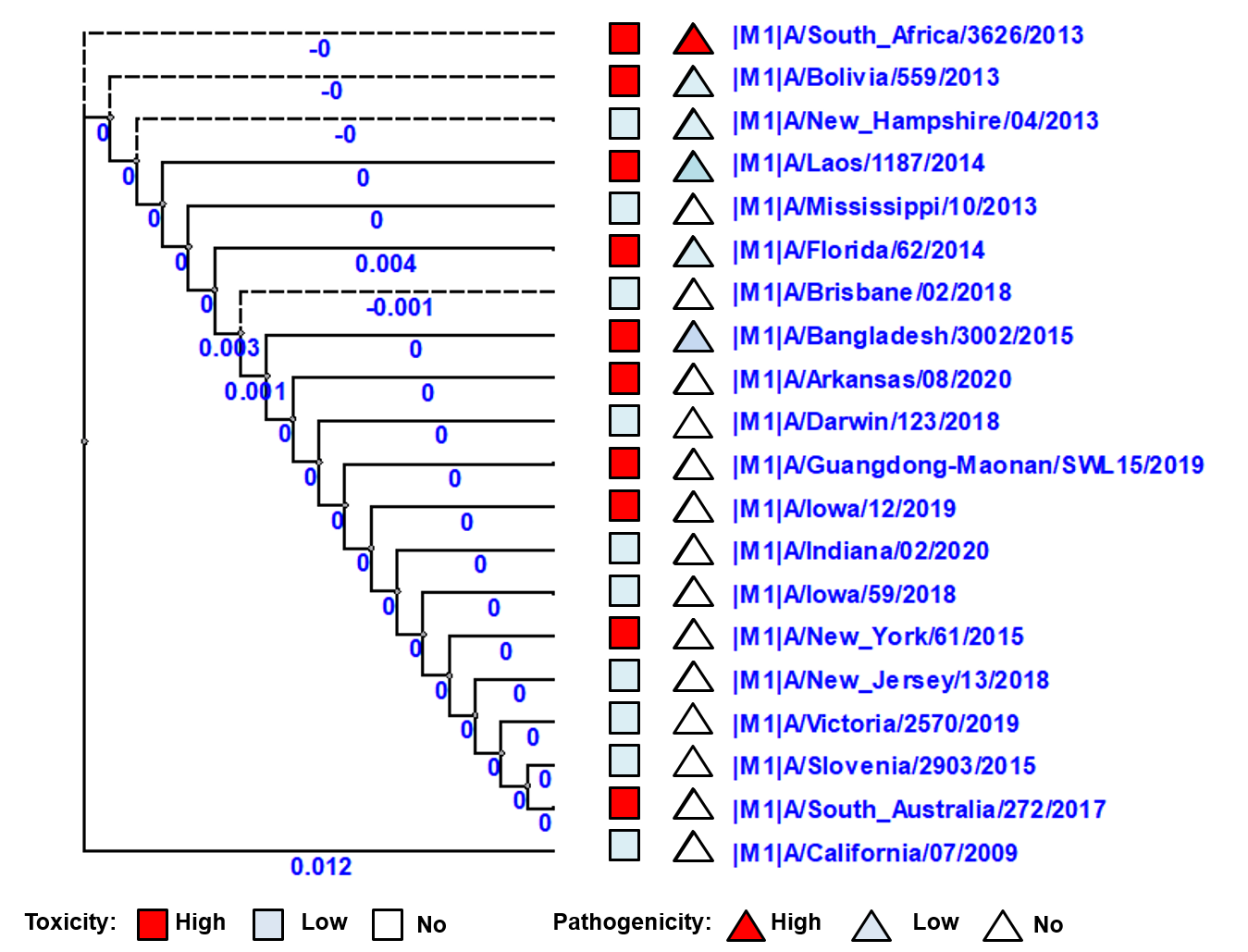

Supplement: Supplementary file 1 [file vaccines-10-00395-s001.zip › vaccines-1522373-supplementary/Figure S5.tif]

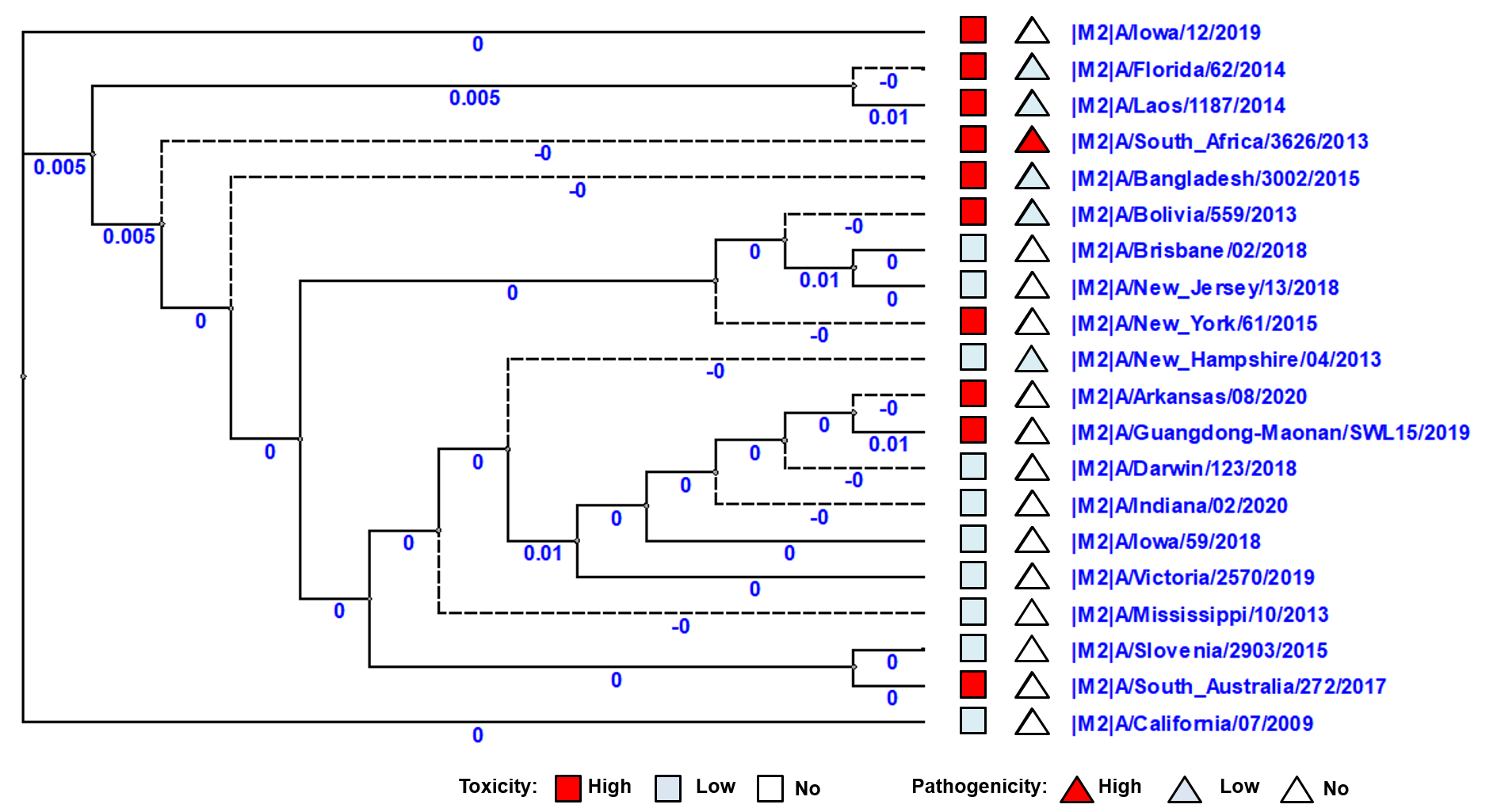

Supplement: Supplementary file 1 [file vaccines-10-00395-s001.zip › vaccines-1522373-supplementary/Figure S6.tif]

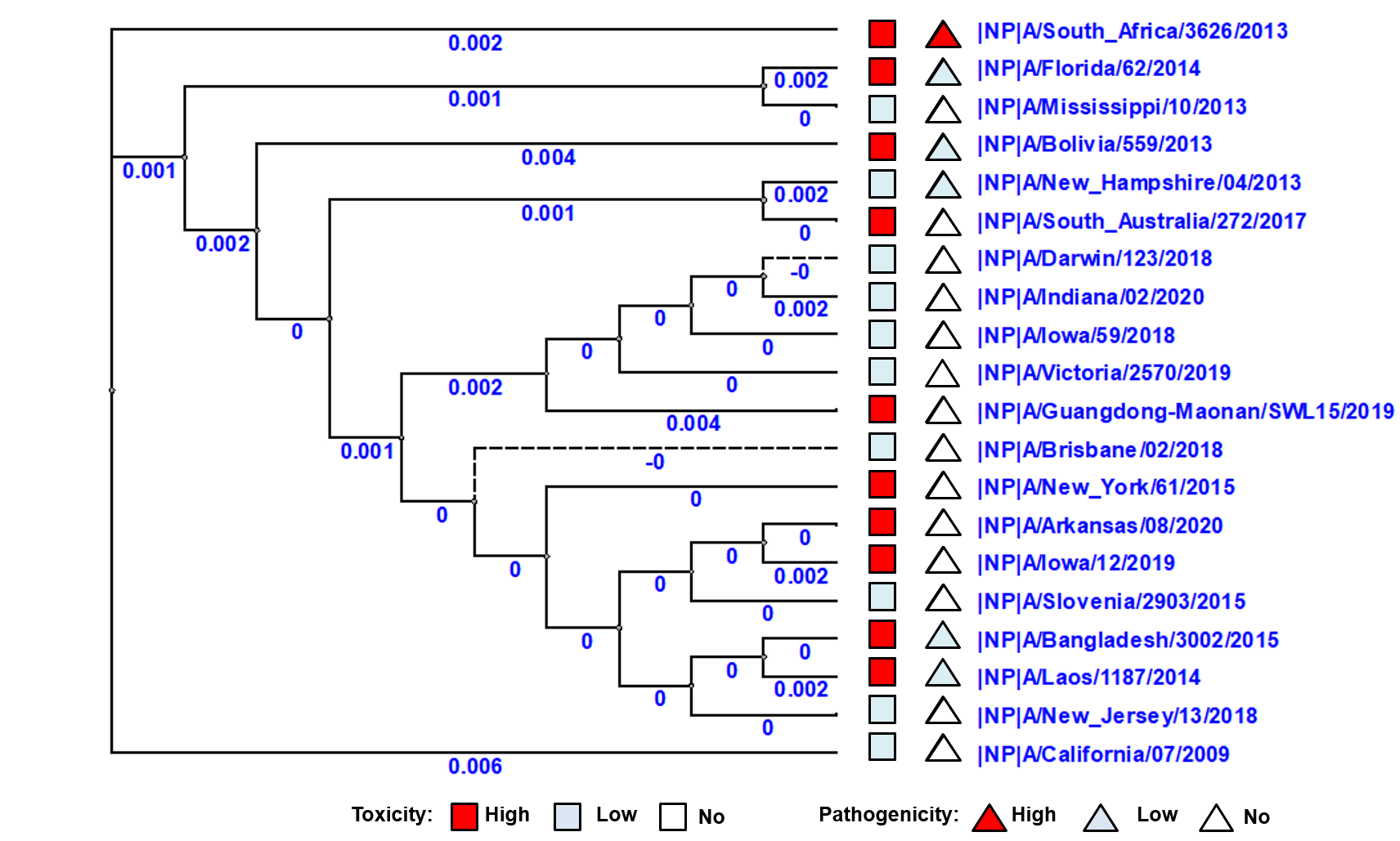

Supplement: Supplementary file 1 [file vaccines-10-00395-s001.zip › vaccines-1522373-supplementary/Figure S7.tif]

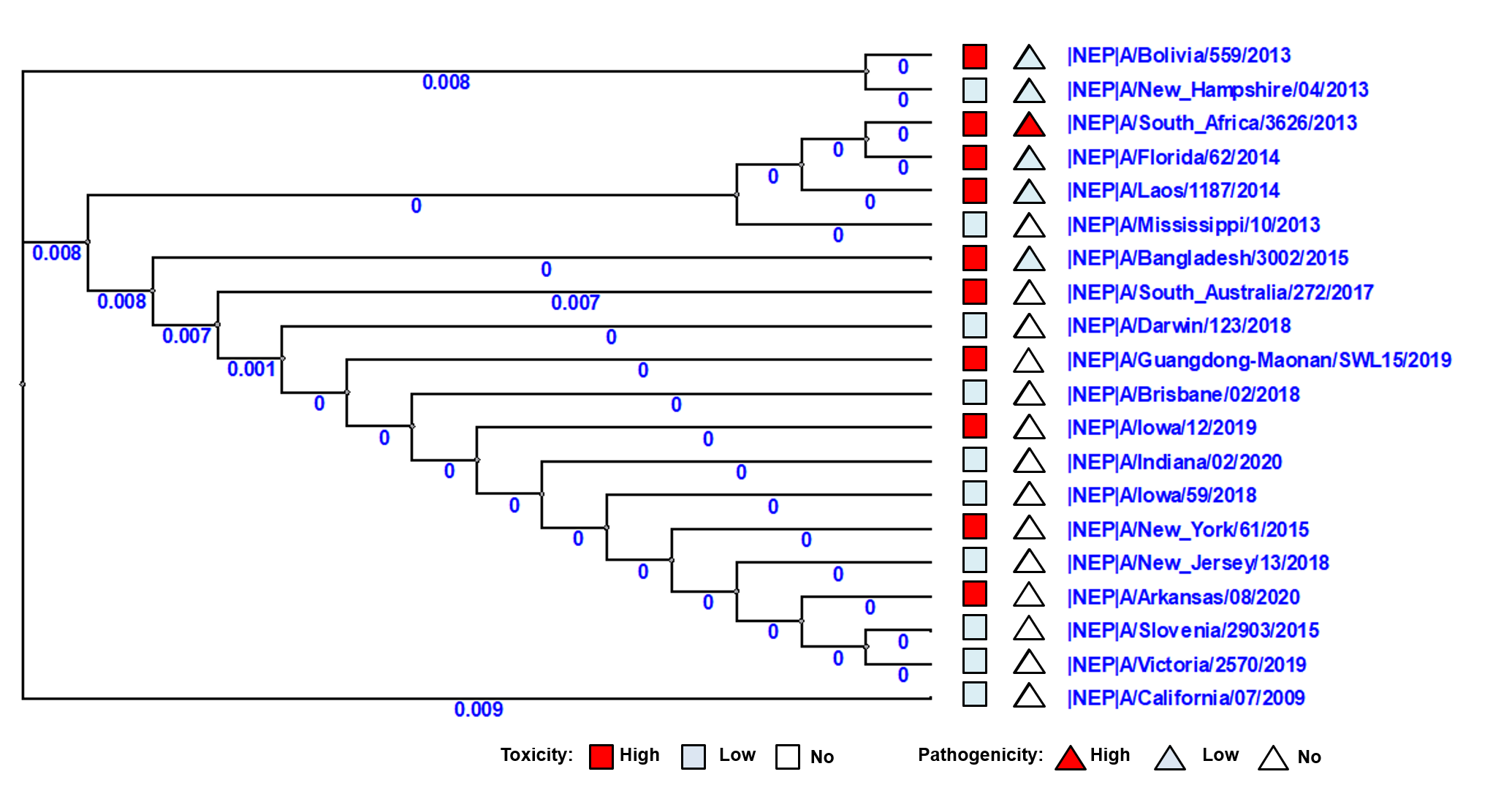

Supplement: Supplementary file 1 [file vaccines-10-00395-s001.zip › vaccines-1522373-supplementary/Figure S8.tif]

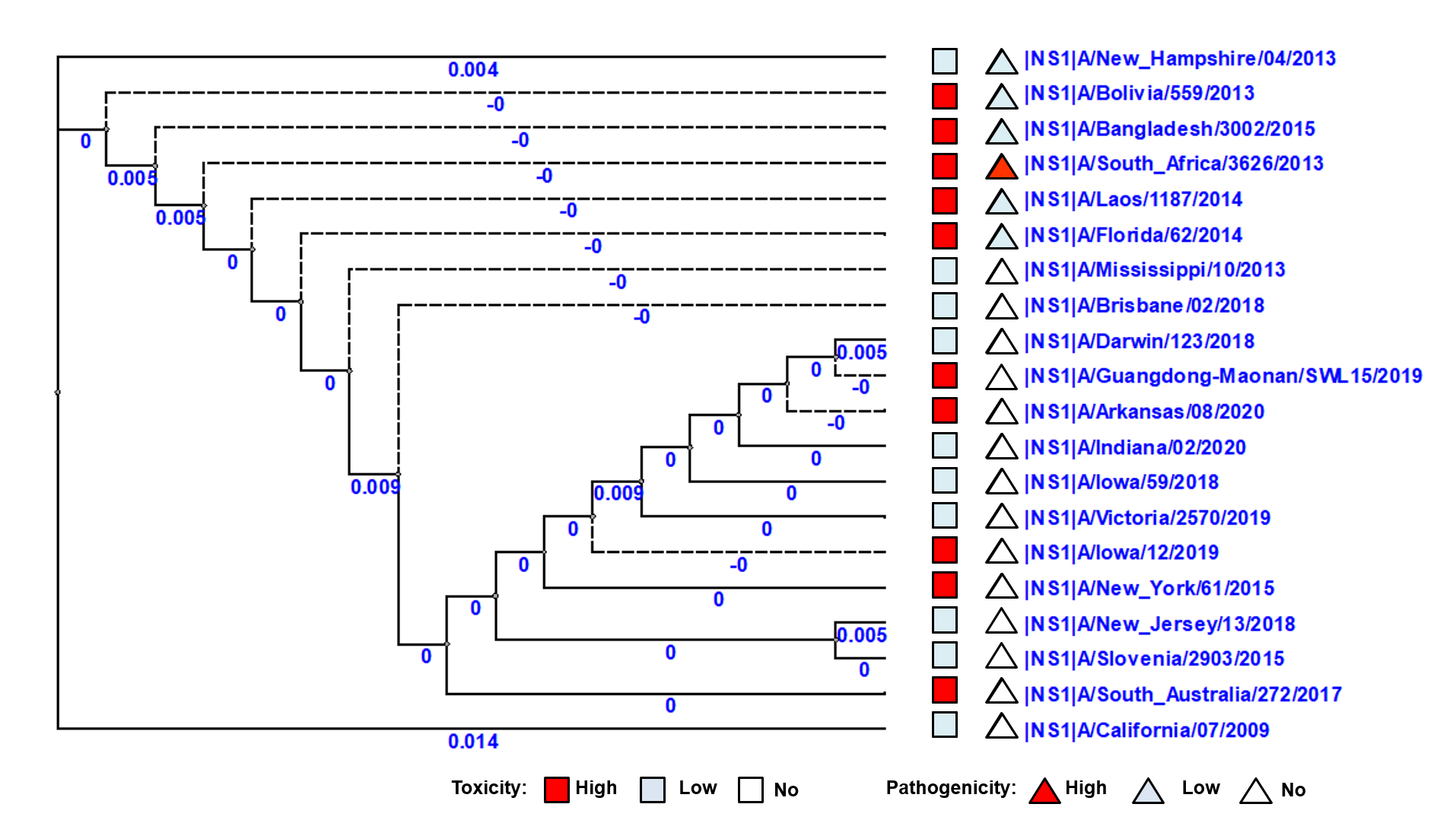

Supplement: Supplementary file 1 [file vaccines-10-00395-s001.zip › vaccines-1522373-supplementary/Figure S9.tif]
